# Supplementary material for: Enhanced tumor response to adoptive T cell therapy with PHD2/3-deficient CD8 T cells
Source: Nat Commun. 2024 Sep 6;15:7789. doi: 10.1038/s41467-024-51782-z (PMC11379939; doi:10.1038/s41467-024-51782-z)
Supplement: Supplementary file 1 — Supplementary Information [file 41467_2024_51782_MOESM1_ESM.pdf]

Enhanced tumour response to adoptive T cell therapy with PHD2/3-deficient CD8 T cells

Tereza Dvorakova[1,2,3], Veronica Finisguerra[1,2,3], Matteo Formenti[1,2,3], Axelle Loriot[1], Loubna Boudhan[1,2,3], Jingjing Zhu [1,2,3,#*], Benoit J Van den Eynde[1,2,3,4,#*]

1. de Duve Institute, UCLouvain, Brussels B-1200, Belgium.
2. Ludwig Institute for Cancer Research, Brussels B- 1200, Belgium.
3. WEL Research Institute, Wavre 1300, Belgium.
4. Ludwig Institute for Cancer Research, Nuffield Department of Clinical Medicine, University of Oxford Oxford, Oxfordshire, UK.

# Equal contributing Authors

*** Corresponding authors :**
benoit.vandeneynde@uclouvain.be and jingjng.zhu@uclouvain.be

Mailing address: Avenue Hippocrate, 75 B1.74.03 – B-1200 Brussels, BELGIUM
Phone number: 0032 2 764 75 72

Supplementary Figure 1

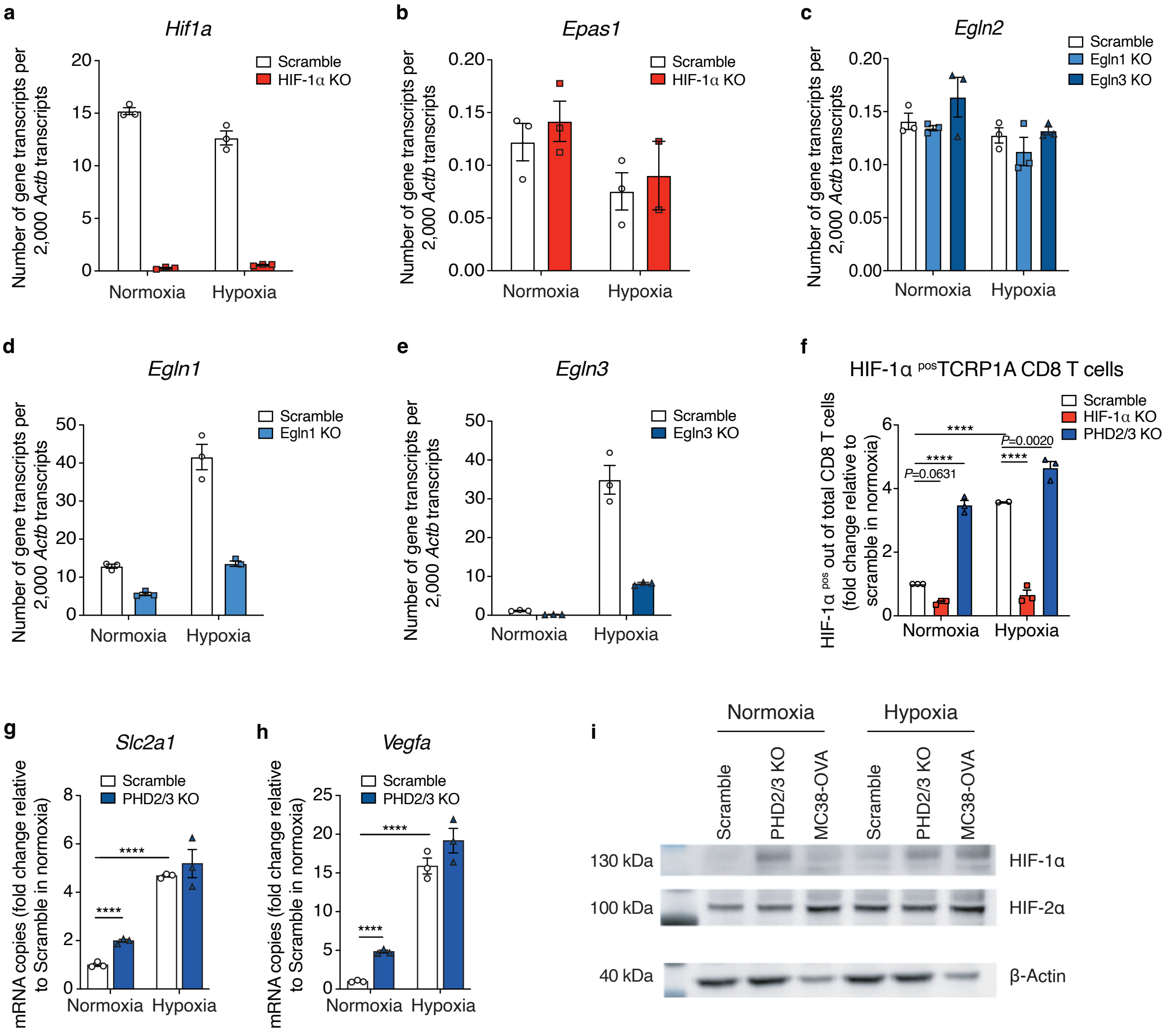

Supplementary Figure 1. Validation of HIF-1α and PHD2/3 knock-out in CD8 T cells

(A-E) RT-qPCR analysis of *Hif1a* (HIF-1α) (A), *Epas* (HIF-2α) (B), *Egln2* (PHD1) (C), *Egln1* (PHD2) (D) and *Egln3* (PHD3) (E),in activated TCRP1A CD8 T cells nucleofected with indicated gRNA-Cas9 complex and incubated under normoxic (21% O2) or hypoxic (1% O2) conditions for 24 hours. The mRNA levels of different genes were measured by quantitative RT-qPCR and normalized to *Actb*. Data are expressed as fold change in mRNA copies, with normalization to scramble T cells cultured under normoxic conditions.
(F) Percentage of HIF-1α-expressing CD8 T cells in activated scramble, HIF-1α KO and PHD2/3 KO TCRP1A CD8 T cells incubated under normoxic (21% O2) or hypoxic (1% O2) conditions for 24 hours, analysed by intracellular flow cytometry staining.
(G-H) RT-qPCR analysis of *Slc2a1* (GLUT1) (G) and *Vegfa* (VEGF) (H) in activated TCRP1A CD8 T cells nucleofected with indicated gRNA-Cas9 complex and incubated under normoxic (21% O2) or hypoxic (1% O2) conditions for 24 hours. The mRNA levels of different genes were measured by quantitative RT-qPCR and normalized to *Actb*. Data are expressed as fold change in mRNA copies, with normalization to scramble T cells cultured under normoxic conditions.
(I) Representative western blot analysis showing HIF-1α, HIF-2α and Beta-Actin (housekeeping) protein levels in nuclear fractions of scramble and PHD2/3 KO TCRP1A CD8 T cells incubated under normoxic (21% O2) or hypoxic (1% O2) conditions for 24 hours. MC38-OVA cells incubated under normoxic or hypoxic conditions were used as blotting controls.
Data are mean ± SEM from one representative out of 3 independent experiments. **** p values <0.0001, calculated by one-way ANOVA with Tukey's multiple comparison test.

Supplementary Figure 2

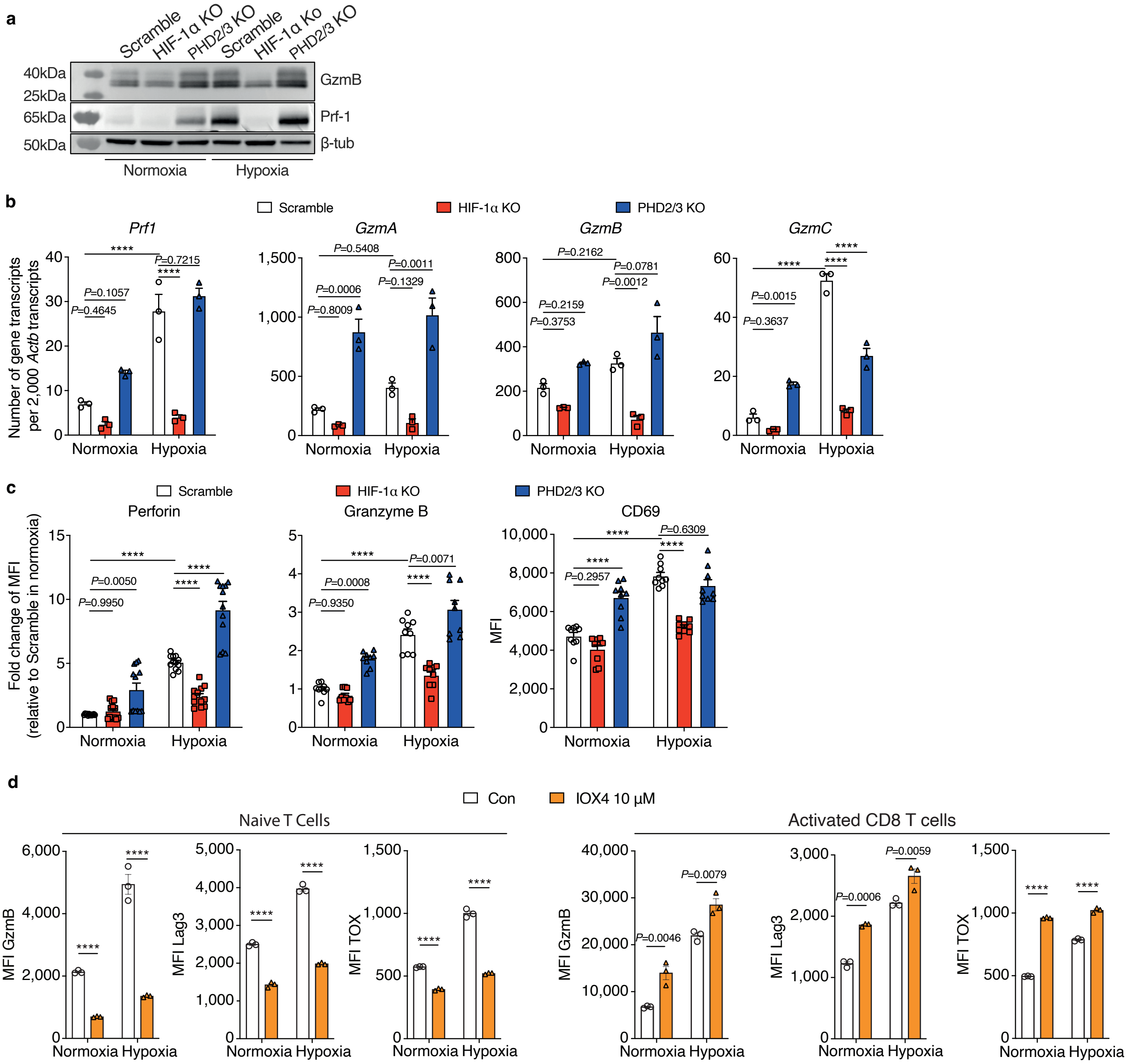

Supplementary Figure 2. Expression of effector molecules in TCRP1A and OT-1 CD8 T cells

(A) Representative Western blot analysis showing Granzyme B and Perforin expression levels in scramble, HIF-1α KO and PHD2/3 KO TCRP1A CD8 T cells incubated under normoxic (21% O2) or hypoxic (1% O2) conditions for 48 hours. Beta-Tubulin was used as housekeeping protein.

(B) RT-qPCR analysis of Perforin (*Prf1*) and Granzymes (*Gzm*) A, B, C gene expression in activated scramble, HIF-1α KO and PHD2/3 KO TCRP1A CD8 T cells incubated under normoxic (21% O2) or hypoxic (1% O2) conditions for 48 hours. The mRNA levels of different genes were measured by quantitative RT-qPCR and normalized to *Actb* (beta-actin). Data are shown as mean ± SEM and are a representative of 3 independent experiments.

(C) Expression of Perforin, Granzyme B and CD69 measured by flow cytometry analysis in Scramble, HIF-1α KO and PHD2/3 KO OT-1 CD8 T cells incubated under normoxic (21% O2) or hypoxic (1% O2) conditions for 48 hours. Data are expressed as fold change in MFI, with normalization to scramble T cells cultured under normoxic conditions.

(D) Flow cytometry analysis of the expression levels of Granzyme B, Tox, and Lag3 on naïve or activated CD8 T cells, treated or not with 10 μM IOX4. Naïve T cells were treated with IOX4 for 3 hours followed by immediate activation, with FACS analysis performed 7 days after activation. Activated T cells were treated with IOX4 4 days after activation, with FACS analysis performed 7 days after activation.

Data are mean ± SEM from one representative experiment out of 3 independent experiments (A, B, D) or a pool of at least 3 independent experiments (C). MFI, median fluorescence intensity. **** p values <0.0001, calculated by one-way ANOVA with Tukey's multiple comparison test.

!"

Supplementary Figure 3

a

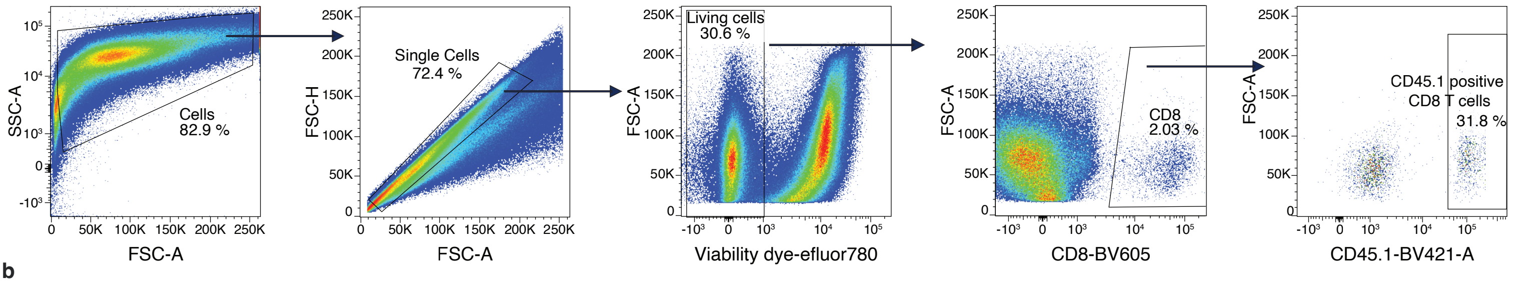

b

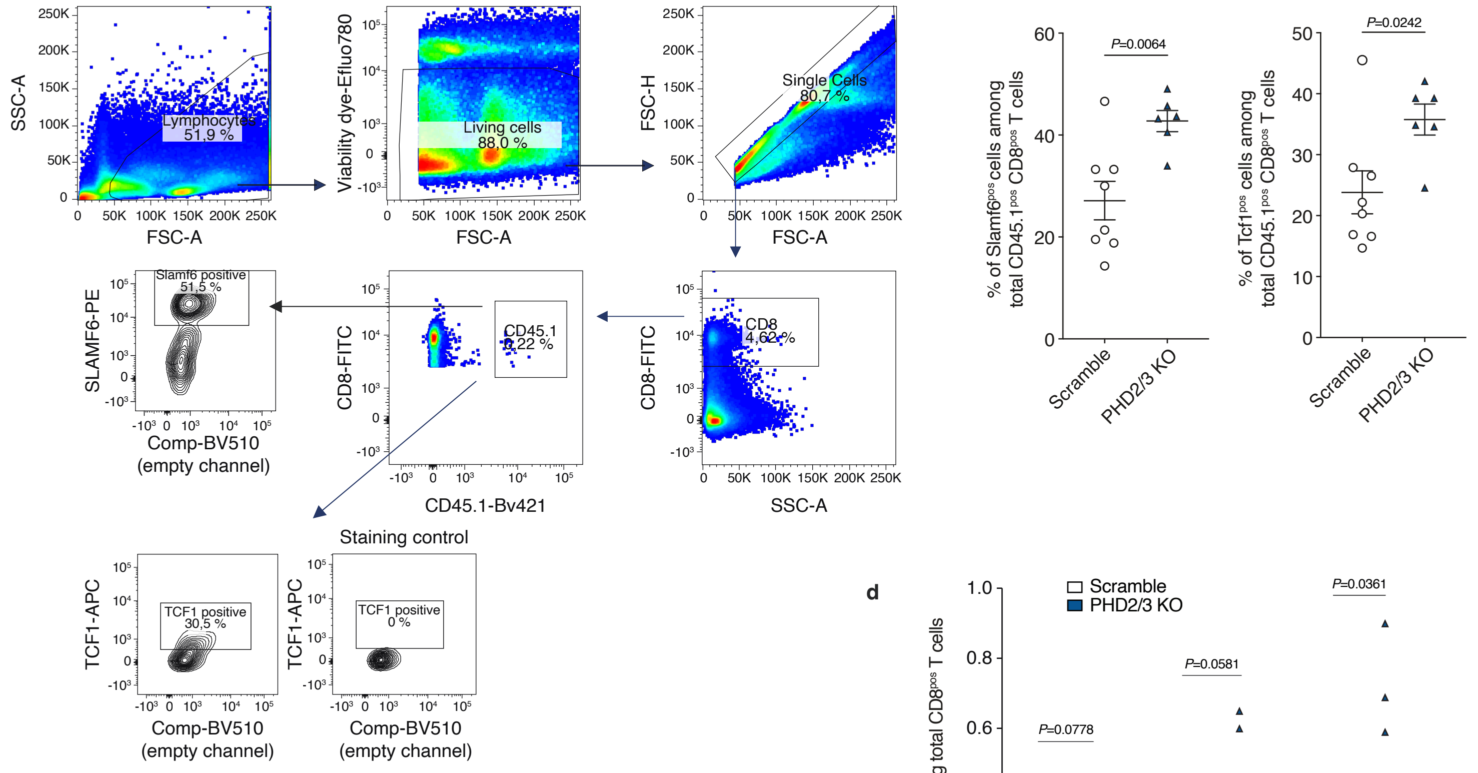

c

	1st tumor implantation (tumor bearing/total)	2nd tumor implantation (tumor bearing/total)
**Scramble**	40% (8/20)	33% (4/12)
**PHD2/3 KO**	0% (0/20)	5% (1/20)

d

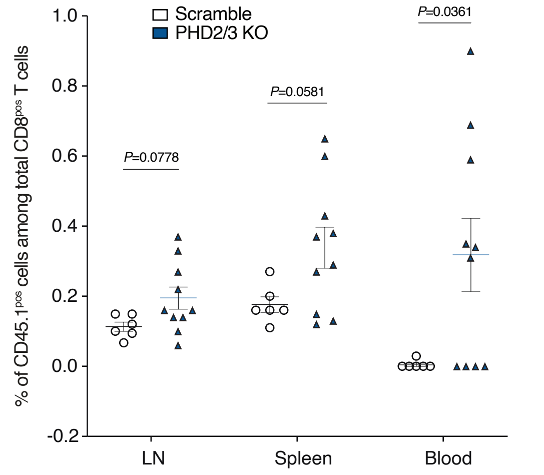

Supplementary Figure 3. The impact of HIF-1α or PHD2/3 deletion in CD8 T cells on their anti-tumor efficacy and memory phenotype

(A) Gating strategy for Figure 4D and 5C.
(B) Flow cytometry analysis was performed to assess the Slamf6^{pos} and Tim3^{pos} populations among CD45.1 positive CD8 T cells within the MC38-OVA tumors of mice that received adoptive-cell transfer of either scramble or PHD2/3 KO OT-1 CD8 T cells.
(C) Concurrent tumor implantation and adoptive-cell transfer were conducted by subcutaneously injecting mice with 1 million MC38-OVA cells and intravenously injecting them with 3 million scramble or PHD2/3 KO CD8 T cells. Tumor formation was monitored for 4 weeks, and mice that developed tumors were recorded. After 4 weeks, mice without tumor were rechallenged subcutaneously with 1 million MC38-OVA tumor cells, and tumor formation was subsequently monitored.
(D) Analysis of the expansion of transferred scramble or PHD2/3 KO CD8 T cells upon tumor-cell rechallenge. Concurrent tumor implantation and adoptive-cell transfer were conducted by subcutaneously injecting mice with 1 million MC38-OVA cells while simultaneously administering an intravenous injection of 3 million scramble or PHD2/3 KO CD8 T cells. After 4 weeks, mice without tumor from each group were intravenously injected with 1 million MC38-OVA tumor cells. Blood, lymph nodes, and spleen samples were collected 24 hours later and analysed for the presence of CD45.1 positive cells.
Data in B and D are mean ± SEM from one experiment. p values were calculated by *t*-tests.

Supplementary table S1

**CRISPR-Cas9 guide crRNAs**	
	**Reference**
Alt-R® CRISPR-Cas9 tracrRNA	1072534
Negative Control crRNA #1	1072544
HIF-1α	Mm.Cas9.HIF1A.1.AA
HIF-1α	Mm.Cas9.HIF1A.1.AC
HIF-1α	Mm.Cas9.HIF1A.1.AD
HIF-2α	Mm.Cas9.EPAS.1.AA
HIF-2α	Mm.Cas9.EPAS.1.AB
PHD2 (Egln1)	Mm.Cas9.EGLN1.1.AA
PHD3 (Egln3)	Mm.Cas9.EGLN3.1.AD
PHD3 (Egln3)	Mm.Cas9.EGLN3.1.AE
PHD3 (Egln3)	Mm.Cas9.EGLN3.1.AF

Supplementary Table S2

**Flow cytometry reagents**		
	**Reference**	**Dilution**
Anti-mCD8α, cl 53-6.7	Biolegend, 100744 (BV605)	1:200
Anti-CD16/CD32 blocking antibody, cl 2.4G2	BD Biosciences, 553141	1:100
Anti-mCD45.1, cl A20	Biolegend, 110732 (BV421)	1:200
Anti-mCD45.2, cl 104	Biolegend, 109808 (PE)	1:200
Anti-mCD69, clH1.2F3	Biolegend, 104514 (APC), 104512(Pe-Cy7)	1:100
Anti-hCD69, cl FN50	Biolegend, 310910 (APC)	1:100
Anti-mCD137, cl 17B5	Biolegend, 106110 (APC)	1:100
Anti-mCD152 (CTLA-4), clUC10-4B9	Biolegend, 106312 (BV421)	1:200
Anti-mCD223 (Lag3), cl C9B7W	Biolegend, 125221 (BV421), 125243 (BV711)	1:50
Anti-mCD279 (PD-1), cl 29F.1A12	Biolegend, 135214 (FITC), 135216 (Pe-Cy7)	1:100
Anti-mCD357 (GITR), cl DTA-1	Biolegend, 126310 (PE)	1:100
Anti-hCD357 (GITR), cl 108-17	Biolegend, 371204 (PE)	1:100
Anti-mCD366 (Tim3), cl B8.2C12	Biolegend, 134008 (APC)	1:100
Anti-m/h Granzyme B, cl GB11	Biolegend, 515406 (AF647), 515408 (Pac Blue)	1:40
Anti-mHIF-1a, clD1S7W	Cell Signaling, 59370 (PE), 52496 (AF647)	1:20
Anti-m perforin, cl S16009A	Biolegend, 154306 (PE), 154304 (APC)	1:40
Anti-h perforin, cl B-D48	Biolegend, 353304 (PE), 353312 (APC)	1:40
Viability dye eFluor780	ThermoFisher Scientific, 65-0865	1:1000
Anti-mCD223 (Lag3), cl C9B7W	Biolegend, 125243 (BV711)	1:200
Anti-Tox, cl 6E6D03	Biolegend, 682604 (Alexa Fluor 594)	1:200
Anti-mLy108 (SLAMF6), cl 330-AJ	Biolegend, 134606 (PE)	1:200

Supplementary table S3

**qRT-PCR assays**	
	**Reference**
CPT1	Mm.PT.58.10147164
HIF-1α	Mm.PT.58.11211292
HIF-2α (Epas1)	Mm.PT.58.13819524
HK2	Mm.PT.58.32698746
Eomes	Mm.PT.58.32983267
MCT1 (Slc16a1)	Mm.PT.58.7462799
PDK1	Mm.PT.58.10680444
PHD1 (Egln2)	Mm.PT.58.31663025
PHD2 (Egln1)	Mm.PT.58.6982418
PHD3 (Egln3)	Mm.PT.58.30654629
Tox	Mm.PT.58.5431010
VEGFA	Mm.PT.58.14200306
SLC2A1	Mm.PT.58.7590689
