# Supplementary material for: Enhanced tumor response to adoptive T cell therapy with PHD2/3-deficient CD8 T cells
Source: Nat Commun. 2024 Sep 6;15:7789. doi: 10.1038/s41467-024-51782-z (PMC11379939; doi:10.1038/s41467-024-51782-z)
Supplement: Supplementary file 3 — Reporting Summary [file 41467_2024_51782_MOESM3_ESM.pdf]

nature portfolio

Corresponding author(s): Benoit J. Van den Eynde, Jingjing Zhu

Last updated by author(s): 20/07/2024

Reporting Summary

Nature Portfolio wishes to improve the reproducibility of the work that we publish. This form provides structure for consistency and transparency in reporting. For further information on Nature Portfolio policies, see our Editorial Policies and the Editorial Policy Checklist.

Statistics

For all statistical analyses, confirm that the following items are present in the figure legend, table legend, main text, or Methods section.

n/a	Confirmed	
☐	☒	The exact sample size (*n*) for each experimental group/condition, given as a discrete number and unit of measurement
☐	☒	A statement on whether measurements were taken from distinct samples or whether the same sample was measured repeatedly
☐	☒	The statistical test(s) used AND whether they are one- or two-sided<br>*Only common tests should be described solely by name; describe more complex techniques in the Methods section.*
☐	☒	A description of all covariates tested
☐	☒	A description of any assumptions or corrections, such as tests of normality and adjustment for multiple comparisons
☐	☒	A full description of the statistical parameters including central tendency (e.g. means) or other basic estimates (e.g. regression coefficient) AND variation (e.g. standard deviation) or associated estimates of uncertainty (e.g. confidence intervals)
☐	☒	For null hypothesis testing, the test statistic (e.g. *F*, *t*, *r*) with confidence intervals, effect sizes, degrees of freedom and *P* value noted<br>*Give P values as exact values whenever suitable.*
☒	☐	For Bayesian analysis, information on the choice of priors and Markov chain Monte Carlo settings
☒	☐	For hierarchical and complex designs, identification of the appropriate level for tests and full reporting of outcomes
☒	☐	Estimates of effect sizes (e.g. Cohen's *d*, Pearson's *r*), indicating how they were calculated

Our web collection on statistics for biologists contains articles on many of the points above.

Software and code

Policy information about availability of computer code

Data collection	RNAseq data were obtained from in-house experiments.
Data analysis	RNAseq was performed on 3 biological replicates for each sample by Macrogen Inc. using Illumina NGS workflow. Fastq files were processed using a standard RNAseq pipeline including Trimmomatic-0.38 to remove low quality reads, hisat2-2.1.0 to align reads to the mouse genome (grcm38), and gene expression levels were evaluated using featureCounts from subread-2.0.0 and Mus_musculus.GRCm38.94.gtf. Differential expression analyses were performed with DESeq2 Bioconductor package v1.38.3. Gene set enrichment analyses were done using clusterProfiler v4.6.2.<br>For in vitro and in vivo data the following softwares were used: Flowjo (version 10.7.1), Graphpad prism (version 9.2) and Microsoft Excel (version 16.76)

For manuscripts utilizing custom algorithms or software that are central to the research but not yet described in published literature, software must be made available to editors and reviewers. We strongly encourage code deposition in a community repository (e.g. GitHub). See the Nature Portfolio guidelines for submitting code & software for further information.

Data

Policy information about availability of data

All manuscripts must include a data availability statement. This statement should provide the following information, where applicable:

- Accession codes, unique identifiers, or web links for publicly available datasets
- A description of any restrictions on data availability
- For clinical datasets or third party data, please ensure that the statement adheres to our policy

All data supporting the findings of this study are available within the Article and its Supplementary Information. RNA-seq data were obtained from in-house experiments. Raw and processed data are available from the Gene Expression Omnibus under accession code GSE271367.

Research involving human participants, their data, or biological material

Policy information about studies with human participants or human data. See also policy information about sex, gender (identity/presentation), and sexual orientation and race, ethnicity and racism.

Reporting on sex and gender	Females and males
Reporting on race, ethnicity, or other socially relevant groupings	/
Population characteristics	Peripheral blood mononuclear cells (PBMCs) were derived from the blood of different haemochromatosis patients, courtesy of Saint-Luc University hospital.
Recruitment	/
Ethics oversight	authorization approval n° CEHF 2021/13SEP/373.

Note that full information on the approval of the study protocol must also be provided in the manuscript.

Field-specific reporting

Please select the one below that is the best fit for your research. If you are not sure, read the appropriate sections before making your selection.

☒ Life sciences ☐ Behavioural & social sciences ☐ Ecological, evolutionary & environmental sciences

For a reference copy of the document with all sections, see nature.com/documents/nr-reporting-summary-flat.pdf

Life sciences study design

All studies must disclose on these points even when the disclosure is negative.

Sample size	No statistical methods were used to determine the sample size. The sample size was chosen based on preliminary experiments and previously published results. The group size for each individual experiment is mentioned in the figure legend.
Data exclusions	There were no deliberate data exclusions.
Replication	Reproducibility of experimental findings (wherever applicable) was verified by either considering analyses of multiple patient samples or equal to at least or more than 3 biologically independent experiments.<br>RNAseq was performed in triplicate as stated earlier.
Randomization	Tumor-bearing mice were randomized at the time when treatment started based on tumor size, and age. For in vitro experiments, no randomization is needed since the cells treated are from the same batch.
Blinding	The tumor measurement was performed with cage labels blinded for treatments. The treatments were performed after the tumor measurement. For in vitro experiments, experiments execution and analysis was performed by different people in the lab.

Reporting for specific materials, systems and methods

We require information from authors about some types of materials, experimental systems and methods used in many studies. Here, indicate whether each material, system or method listed is relevant to your study. If you are not sure if a list item applies to your research, read the appropriate section before selecting a response.

Materials & experimental systems

n/a	Involved in the study	
☐	☒	Antibodies
☐	☒	Eukaryotic cell lines
☒	☐	Palaeontology and archaeology
☐	☒	Animals and other organisms
☒	☐	Clinical data
☒	☐	Dual use research of concern
☒	☐	Plants

Methods

n/a	Involved in the study	
☒	☐	ChIP-seq
☐	☒	Flow cytometry
☒	☐	MRI-based neuroimaging

Antibodies

Antibodies used

Flow cytometry:
Anti-mCD8α, cl 53-6.7, Biolegend, 100744 (BV605), 1:200 dilution.
Anti-CD16/CD32 blocking antibody, cl 2.4G2, BD Biosciences, 553141, 1:1000 dilution.
Anti-mCD45.1, cl A20 Biolegend, 110732 (BV421), 1:50 dilution.
Anti-mCD45.2, cl 104 Biolegend, 109808 (PE), 1:100 dilution.
Anti-mCD69, clH1.2F3 Biolegend, 104514 (APC), 104512(Pe-Cy7), 1:100 dilution.
Anti-hCD69, cl FN50 Biolegend, 310910 (APC), 1:100 dilution.
Anti-mCD137, cl 17B5 Biolegend, 106110 (APC), 1:100 dilution.
Anti-mCD152 (CTLA-4), clUC10-4B9 Biolegend, 106312 (BV421), 1:50 dilution.
Anti-mCD178 (FasL), cl Kay-10 Biolegend, 106805 (PE), 1:50 dilution.
Anti-mCD223 (Lag3), cl C9B7W Biolegend, 125221 (BV421), 1:100 dilution.
Anti-mCD279 (PD-1), cl 29F.1A12 Biolegend, 135214 (FITC), 135216 (Pe-Cy7), 1:100 dilution.
Anti-mCD357 (GITR), cl DTA-1 Biolegend, 126310 (PE), 1:100 dilution.
Anti-hCD357 (GITR), cl 108-17 Biolegend, 371204 (PE), 1:50 dilution.
Anti-mCD366 (Tim3), cl B8.2C12 Biolegend, 134008 (APC), 1:100 dilution.
Anti-m/h Granzyme B, cl GB11 Biolegend, 515406 (AF647), 515408 (Pac Blue), 1:40 dilution.
Anti-mHIF1a, clD1S7W Cell Signaling, 59370 (PE), 52496 (AF647), 1:50 dilution.
Anti-m perforin, cl S16009A Biolegend, 154306 (PE), 154304 (APC), 1:40 dilution.
Anti-h perforin, cl B-D48 Biolegend, 353304 (PE), 353312 (APC), 1:50 dilution.
anti-m/h Granzyme B, cl GB11 Biolegend, 515408 (Pac Blue), 1:200 dilution.
Anti-mCD223 (Lag3), cl C9B7W Biolegend, 125243 (BV711), 1:200 dilution.
Anti-Tox, cl 6E6D03 Biolegend, 682604 (Alexa Fluor 594), 1:200 dilution.
Anti-mLy108 (SLAMF6), cl 330-AJ Biolegend, 134606 (PE), 1:200 dilution.

Western blot:
anti-HIF-1α, Cayman chemicals, 10006421, 1:3000 dilution
anti-granzyme B, Abcam, ab255598, 1:1000 dilution.
anti-perforin, Cell signaling, 31647, 1:1000 dilution.
HRP-anti-HDAC1, Cell signaling, 59581, 1:5000 dilution.
HRP-anti-GAPDH, Cell signaling, 8884S, 1:3000 dilution.
HRP-b-tubulin, Cell signaling, 5346S, 1:3000 dilution.
anti-HIF2a, Novus Biologicals, NB100-122, 1:1000 dilution

Validation

All antibodies are validated by the manufacturer.

Eukaryotic cell lines

Policy information about cell lines and Sex and Gender in Research

Cell line source(s)

T429.11: a cell line derived from induced TiRP melanoma tumor model in the lab (Zhu, J. et al. Resistance to cancer immunotherapy mediated by apoptosis of tumor-infiltrating lymphocytes. Nat Commun 8, 1404 (2017))

MC38-OVA: cell line derived from MC38 as described here (Petit, P. F. et al. T Cell-Mediated Targeted Delivery of Anti-PD-L1 Nanobody Overcomes Poor Antibody Penetration and Improves PD-L1 Blocking at the Tumor Site. Cancer Immunol Res 10, 713-727 (2022))

LL2-Thy1.1-OVA (LLC-OVA) cells, expressing a cytoplasmic form of ovalbumin, were a kind gift from D. Fearon (Cancer research UK Cambridge Institute).

L1210.P1A.B7-1, T429.11, P511 and P1.204 cell lines were described here: (Zhu, J. et al. Resistance to cancer immunotherapy mediated by apoptosis of tumor-infiltrating lymphocytes. Nat Commun 8, 1404 (2017))

Authentication

The cell authentication for all murine cell lines was performed by ATCC cell line authentication service using short tandem repeat (STR) profiling in 2022.

Mycoplasma contamination	All cell lines used were regularly tested (every one month and half) and are negative for mycoplasma
Commonly misidentified lines (See ICLAC register)	no

Animals and other research organisms

Policy information about studies involving animals; ARRIVE guidelines recommended for reporting animal research, and Sex and Gender in Research

Laboratory animals	All mice were housed at an ambient temperature around 21-23 C°, humidity of 40-60% and a light dark cycle of 12 hours.<br>TiRP mice: TiRP mice have been created by crossing Ink4a/Arfflox/flox mice with mice carrying a transgenic construct controlled by the tyrosinase promoter and driving the expression of H-Ras12V and Trap1a which encodes a MAGE-type tumor antigen P1A. Females mice with age between 10-18 weeks were used. (previosuly described here : Zhu, J. et al. Resistance to cancer immunotherapy mediated by apoptosis of tumor-infiltrating lymphocytes. Nat Commun 8, 1404 (2017)).<br><br>TCR-P1A mice: TCRP1A mice heterozygous for the H 2Ld/P1A35-43-specific TCR transgene were kept on the B10.D2;Rag1−/− background, only female mice between age 9-12 were used (previosuly described here : Zhu, J. et al. Resistance to cancer immunotherapy mediated by apoptosis of tumor-infiltrating lymphocytes. Nat Commun 8, 1404 (2017)).<br><br>TCR-OT-I mice:Only female mice between age 9-12 were used. CD57BL/6-Tg(Tcra Tcrb)1100Mjb/Crl (OT-1 OVA-TCR) were bred with B6.SJL-Ptprca Pepcb/BoyCrl mice (both purchased from Charles River) to generate heterozygous CD45.1pos and CD45.2pos OT-1 OVA-TCR mice.<br><br>C57BL/6J mice: Female mice with age between 6-12 weeks were used.
Wild animals	no
Reporting on sex	Female mice (aged 9–12 weeks) were used for CD8 T cell isolation and for in vivo tumor transplantation to avoid the rejection of male-specific antigens that could occur when using male mice.
Field-collected samples	no
Ethics oversight	Mice were maintained under specific pathogen-free conditions at the Platform Laboratory Animal Facility of the de Duve Institute. Housing and experimental procedures were approved by the Animal Ethical Committee of the UCLouvain (2015/UCL/MD/015; 2019/UCL/MD/018) and mice were euthanized if a humane endpoint was reached.

Note that full information on the approval of the study protocol must also be provided in the manuscript.

Flow Cytometry

Plots

Confirm that:

☒ The axis labels state the marker and fluorochrome used (e.g. CD4-FITC).

☒ The axis scales are clearly visible. Include numbers along axes only for bottom left plot of group (a 'group' is an analysis of identical markers).

☒ All plots are contour plots with outliers or pseudocolor plots.

☒ A numerical value for number of cells or percentage (with statistics) is provided.

Methodology

Sample preparation	Tumors and spleens were isolated from tumor-bearing mice. The tumor samples were dissociated by using a cocktail of three enzymes (Collagenase I, Collagenase II and Dispase) with the help of gentle macs dissociator. Spleens were gently smashed in cell culture medium to generate cell suspension. Single cells were obtained by filtration of tissue homogenate through a 40-uM filter. Spleen samples were simply grinded gently with the flat end of a syringe in full T cell culture medium. The cell suspensions were collected and filtered through a 70 micrometer filter (#352350, FALCON).
Instrument	BD LSRFortessa, BD FACSVerse
Software	FlowJo 10.7.1
Cell population abundance	Minimal cell number in target gate was 1000.
Gating strategy	Gating strategy on tumors<br>1. FSC and SSC<br>2. FSC-H and FSC-A to gate on single cell population<br>3. Viability 780 to gate on live cells, this is defined as Viability 780 negative cells.<br>4. CD45.2+ cells

5. CD8+ cells for T cells,
6. CD45.1+ cells for adoptively transferred cells
7. Granzyme B, Perforin, CD69 expressions were measured in CD45.1+ population
The threshold were placed based on fluorescence minus one (FMO) controls.

☒ Tick this box to confirm that a figure exemplifying the gating strategy is provided in the Supplementary Information.
