# Supplementary material for: Enhanced tumor response to adoptive T cell therapy with PHD2/3-deficient CD8 T cells
Source: Nat Commun. 2024 Sep 6;15:7789. doi: 10.1038/s41467-024-51782-z (PMC11379939; doi:10.1038/s41467-024-51782-z)

Enhanced tumour response to adoptive T cell therapy with PHD2/3-deficient CD8 T cells

Tereza Dvorakova[1,2,3], Veronica Finisguerra[1,2,3], Matteo Formenti[1,2,3], Axelle Loriot[1], Loubna Boudhan[1,2,3], Jingjing Zhu [1,2,3,#*], Benoit J Van den Eynde[1,2,3,4,#*]

1. de Duve Institute, UCLouvain, Brussels B-1200, Belgium.
2. Ludwig Institute for Cancer Research, Brussels B- 1200, Belgium.
3. WEL Research Institute, Wavre 1300, Belgium.
4. Ludwig Institute for Cancer Research Nuffield Department of Clinical Medicine, University of Oxford Oxford, Oxfordshire, UK.

# Equal contributing Authors

*** Corresponding authors :**
benoit.vandeneynde@uclouvain.be and jingjng.zhu@uclouvain.be

Mailing address: Avenue Hippocrate, 75 B1.74.03 – B-1200 Brussels, BELGIUM
Phone number: 0032 2 764 75 72

Immunoblots for Figure 1b

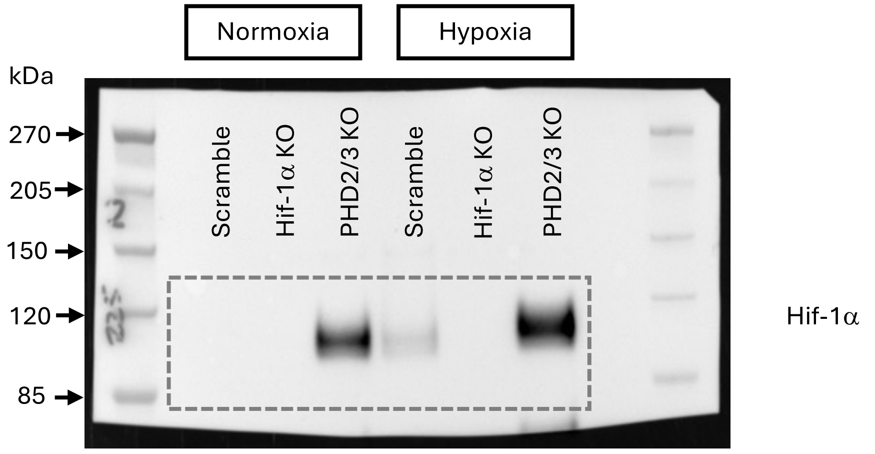

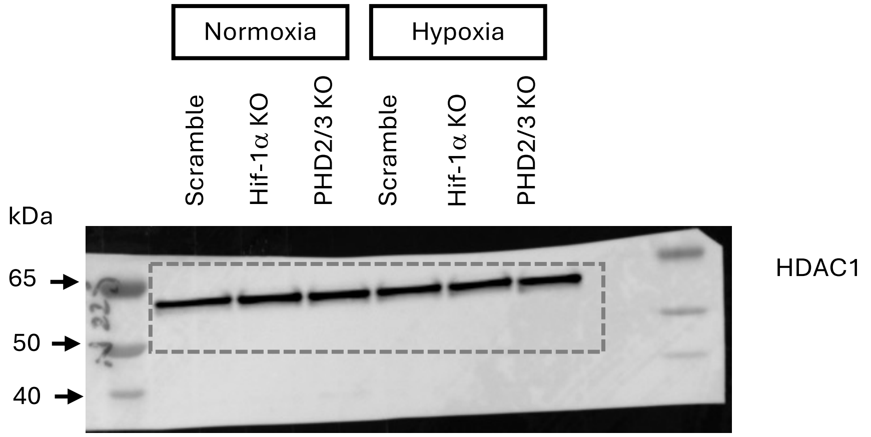

Immunoblots for Figure 3c

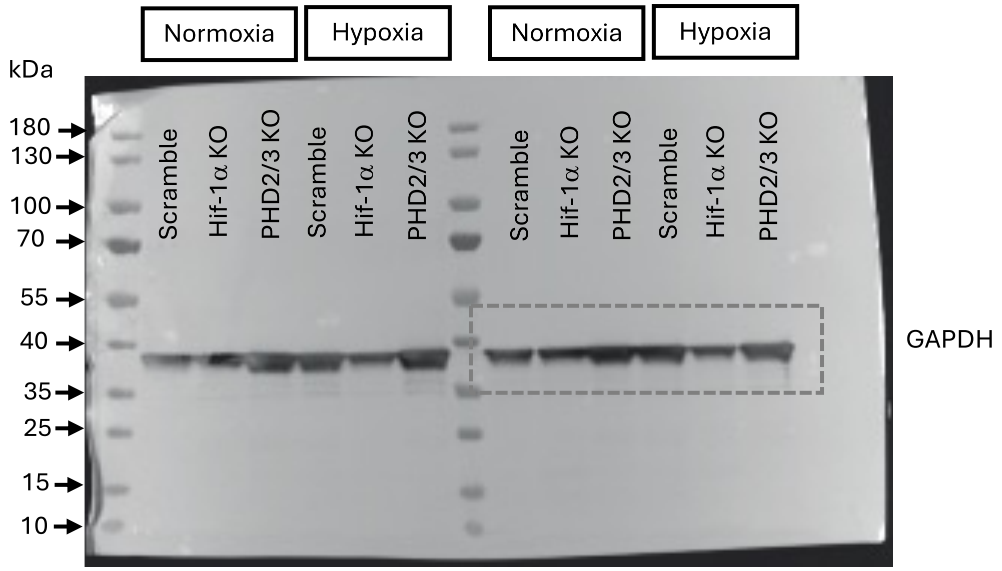

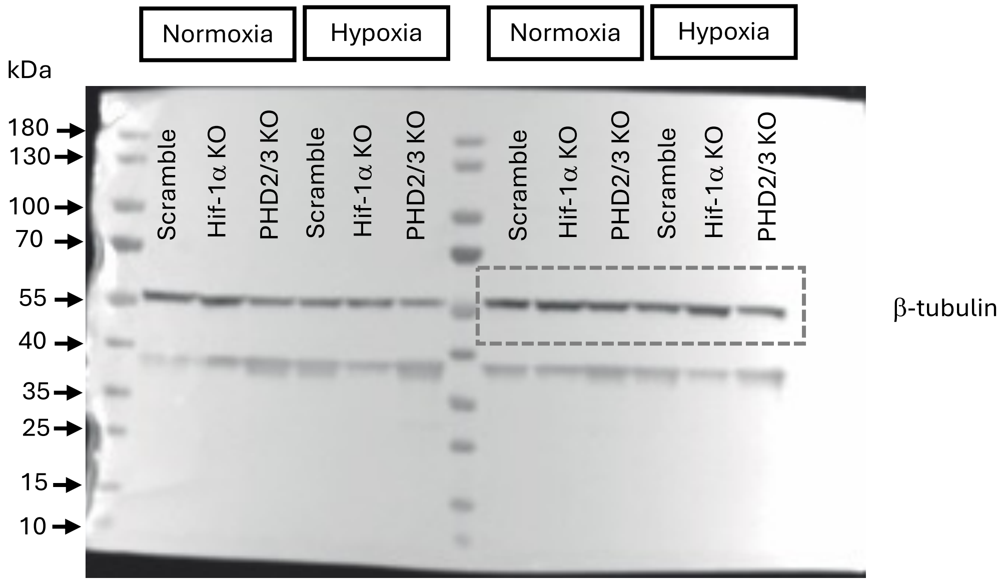

Immunoblots for Supplementary Figure 2a

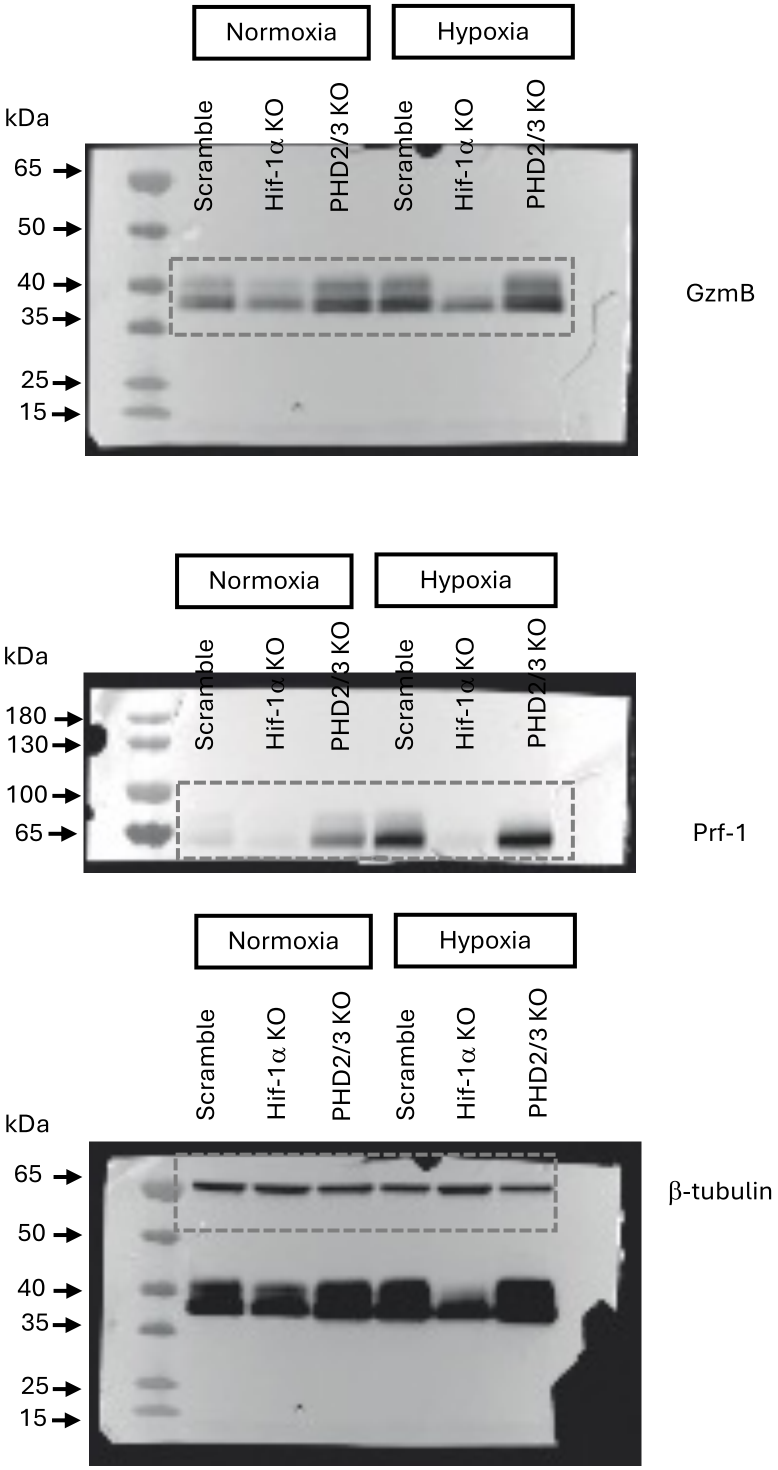

Supplement: Supplementary file 4 — Source Data [file 41467_2024_51782_MOESM4_ESM.zip › Source Data file [38].pdf]
